# Formation of Zirconium Hydrophosphate Nanoparticles and Their Effect on Sorption of Uranyl Cations

**DOI:** 10.1186/s11671-017-1987-y

**Published:** 2017-03-21

**Authors:** Nataliya Perlova, Yuliya Dzyazko, Olga Perlova, Alexey Palchik, Valentina Sazonova

**Affiliations:** 1 Department of Physical and Colloid Chemistry, Odessa I. I. Mechnikov National University of the MES of Ukraine, Dvoryanska str., 2, Odesa, 65082 Ukraine; 2 Department of Sorption and Membrane Materials and Processes, V.I. Vernadskii Institute of General and Inorganic Chemistry of the NAS of Ukraine, Palladin ave. 32/34, Kyiv, 03142 Ukraine

**Keywords:** Organic-inorganic ion-exchanger, Composite, Nanoparticles, Zirconium hydrophosphate, Uranium

## Abstract

Organic-inorganic ion-exchangers were obtained by incorporation of zirconium hydrophosphate into gel-like strongly acidic polymer matrix by means of precipitation from the solution of zirconium oxychloride with phosphoric acid. The approach for purposeful control of a size of the incorporated particles has been developed based on Ostwald-Freundich equation. This equation has been adapted for precipitation in ion exchange materials. Both single nanoparticles (2–20 nm) and their aggregates were found in the polymer. Regulation of salt or acid concentration allows us to decrease size of the aggregates approximately in 10 times. Smaller particles are formed in the resin, which possess lower exchange capacity. Sorption of U(VI) cations from the solution containing also hydrochloride acid was studied. Exchange capacity of the composites is ≈2 times higher in comparison with the pristine resin. The organic-inorganic sorbents show higher sorption rate despite chemical interaction of sorbed ions with functional groups of the inorganic constituent: the models of reaction of pseudo-first or pseudo-second order can be applied. In general, decreasing in size of incorporated particles provides acceleration of ion exchange. The composites can be regenerated completely, this gives a possibility of their multiple use.

## Background

Due to unique nuclear properties, uranium is used not only for military demands but also for civilian needs, manly as a fuel for nuclear power plants. Theoretically, about 2 × 10^13^ J of energy can be produced by 1 kg of uranium-235, this is an equivalent of 1.5 × 10^6^ kg of coal [1, 2]. Other fields of uranium application are geology and aerospace industry. Uranium is also used as a fluorescence colorant in uranium glasses.

Except traditional uranium-containing ores, such minerals as autunite [3], parsonsite [4], and monazite [5] can be a source of uranium [6], particularly deposit of monazite is located along the Sea of Azov in Ukraine [7]. Processing of monazite involves hydrochloride acid [8], in media of which uranium (VI) exists in cationic forms [9]. The problem of waste utilization arises, since the maximal permissible concentration for soluble U(VI) compounds in water is 0.015 mg dm^−3^, but even lower values are recommended [10]. The requirements are so strict due to both radioactivity and chemical toxicity of uranium (toxicity is more dangerous than radioactivity). Uranium and decay products inflect all organs and tissues of living organisms.

In order to decrease the U(VI) content in liquid wastes down to maximal permissible concentration, chemical [11] or photocatalytic [12] reduction of soluble U(VI) compounds can be carried out. Insoluble UO_2_ is formed by this manner. These methods require considerable amounts of reagents, so the problem of secondary water pollution must be solved. Similar problem is characteristic for ultrafiltration enhanced with polyelectrolytes [13]. Reverse osmosis [14] is attractive for neutral solution since the membranes, as well as ultrafiltration separators, can be damaged in acidic media.

Ion exchange and adsorption are considered as the most attractive methods for U(VI) removal from diluted solutions, particularly for tertiary treatment of water [15]. Currently, composite sorbents are considered as the most attractive materials. They have to combine such properties as significant exchange capacity, selectivity, high rate of sorption, and facile regeneration. Additionally, the sorbents should be in a form of granules in order to provide their usage in columns. Development of the materials that possess the necessary complex of properties is the actual task.

Such composites as zirconium-antimony oxide/polyacrylonitrile [16], graphene-based materials [17–22], sorbents containing magnetic particles (iron, Fe_3_O_4_, and CoFe_2_O_4_) [22–27], biomaterials [28], polysulfide/layered double hydroxides [29], materials containing carbon nanotubes [25, 30], clay [31], or biopolymers [26] were proposed. Some mentioned sorbents are mechanically instable (carbon-based sorbents), some of them are destroyed in strongly acidic media during regeneration (magnetic sorbents and biomaterials). If the composite is based on inert polymer [16], its sorption capacity is insignificant. Some types of composites are suitable only for anion removal [32, 33].

Commercial strongly acidic ion-exchange resins are characterized by high sorption rate [34, 35]. However, their selectivity is low. Alternately, weakly acidic [36] or chelate [37] resins show considerable selectivity towards U(VI). At the same time, sorption on these materials is slow. In order to improve selectivity of strongly acidic resins towards Ni^2+^, Cd^2+^, and Pb^2+^, they were modified with zirconium hydrophosphate (ZHP) [38–44]. The inorganic sorbent shows high selectivity towards toxic cations [45, 46], particularly U(VI) [46, 47] due to formation of complexes with functional groups [48]. Deposition of insoluble U(VI) compounds in ZHP pores is also suggested [46]. Furthermore, ZHP provides better selectivity of ion exchange membranes towards hardness ions [49]. The ion-exchanger was also applied to modification of track membrane in order to enhance its stability against fouling with organics [50].

The rate of ion exchange on the composites based on ion exchange resin depends on size of incorporated ZHP particles [38, 39, 41]. No sufficient deterioration of sorption rate was found for the composites containing non-aggregated nanoparticles [38, 39]. At the same time, particles of micron size slow down ion exchange [41]. The particle size can be controlled during the resin modification taking the Ostwald-Freundlich equation into consideration [42]. This equation was developed for precipitation from free solutions [51]. When the inorganic constituent is precipitated in ion exchange polymer, the properties of the matrix have to be considered.

Thus, the aim of the investigation is to develop the composites for removal of U(VI) cations from acidic aqueous solutions produced during monazite processing. The tasks involve adaptation of the Ostwald-Freundlich equation to modification of ion exchange matrix, experimental verification of the theoretical approach, investigation of phosphorus state in ZHP and testing of the samples.

## Experimental

### Modification of Ion Exchange Resin

*Dowex HCR-S* strongly acidic gel-like cation exchange resin (Dow Chemical) was chosen for investigations. This material, which contains ≈8% of cross-linking agent (divinylbenzene, DVB), is characterized by the highest mobility of sorbed ions among analogues [52]. A flexible gel-like resin (*Dowex WX-2*. 2% DVB) was also studied for comparison. This type of resins is usually used for electromembrane removal of divalent cations from aqueous solutions [42, 45].

The resins (polymer ion exchange matrices) were modified with amorphous ZHP. In comparison with hydrated oxides and phosphates of other metals, this material is characterized by chemical stability, particularly against hydrolysis. In opposite to crystalline modifications, amorphous ZHP can be easy regenerated.

The modification procedure involved impregnation of the resins with ZrOCl_2_ solution followed by treatment with H_3_PO_4_ solution at 298 K. A ratio of volumes of solid and liquid phases was 1:100. In some cases, additionally sorbed ZrOCl_2_ electrolyte was removed from the resin by washing with 0.01 M HCl solution before ZHP precipitation. Marking of the samples as well as the modification conditions, which were varied in opposite to [38–42], are given in Table 1.

**Table 1:** Modification of ion exchange resins with ZHP

Marking	Polymer matrix	Concentration of a ZrOCl_2_ solution, M	Washing with a HCl solution	Concentration of a H_3_PO_4_ solution, M	Particle size, mm
*CR-1*	*Dowex HCR-S*	1.00	–	1.00	0.45
*CR-2*	*Dowex WX-2*	1.00	–	1.00	–
*CR-3*	*Dowex HCR-S*	1.00	Washing	1.00	0.31
*CR-4*	*Dowex HCR-S*	1.00	Washing	0.10	0.45
*CR-5*	*Dowex HCR-S*	1.00	Washing	0.01	0.40
*CR-6*	*Dowex HCR-S*	0.30	Washing	1.00	0.32
*CR-7*	*Dowex HCR-S*	0.10	Washing	1.00	0.39
*CR-8*	*Dowex HCR-S*	0.01	Washing	1.00	0.41

After precipitation, the samples were washed with deionized water up to pH 7 of the effluent; dried under vacuum conditions at 343 K down to constant mass, treated with ultrasound at 30 kHz using *Bandelin* ultrasonic bath (Bandelin, Hungary), and dried in a desiccator over CaCl_2_ at 293 K.

### Determination of Grain Size and Visualization of Incorporated Particles

In each case, sizes of 300 grains were determined using Crystal-45 optical microscope (Konus, USA). The data for dominant particles are given in Table 1.

Before investigations of morphology of the composites, the samples were grinded and treated with ultrasound. *JEOL JEM 1230* transmission electron microscope (Jeol, Japan) was used for visualization of ZHP particles.

### NMR Spectroscopy

The samples were inserted into the tube with a diameter of 5 mm, NMR ^31^P spectra were measured with *AVANCE 400* spectrometer (Bruker, Germany) using single-pulse technique under the accumulation mode at 162 MHz. Chemical shift was determined relatively to 85% H_3_P0_4_.

### Sorption and Desorption of U(VI) Compounds Under Batch Conditions

The ion-exchangers based on *Dowex HCR-S* resin were applied to investigations since the resins containing 8% DVB are traditionally used for ion exchange processes. The experiments were carried out at 298 K. UO_2_Ac_2_·2H_2_O salt (Chemapol, Czech Republic) was used for preparation of solutions. The solutions contained also HCl (0.02 M, pH 2.5), which is applied to monazite processing [8].

The solution with initial U(VI) concentration of 2 × 10^−4^ M was used for study of sorption rate. A series of weighted air-dry samples (0.1 g) were prepared and inserted to flasks, then deionized water was added. After swelling, water was removed and the solution (50 cm^3^) was added. The content of the flasks was stirred by means of *Water Bath Shaker Type 357* (Elpan, Poland). After a predetermined time, the solid and liquid from one flask were separated; after the next period, the solution was removed from the second flask. U(VI) was determined in a form of complexes with Arsenazo III: the solution was analyzed using *Shimadzu UV-mini1240* spectrophotometer (Shimadzu, Japan) at 670 nm [53].

The degree of uranium removal (sorption degree), *S*, was calculated as $\frac{C_i-{C}_t}{C_i}\times 100\%$, where *C*_*i*_ and *C*_*t*_ are the initial concentration and concentration after certain time, respectively. Sorption capacity (*A*) was determined as $\frac{V\left({C}_i-{C}_t\right)}{m}$, where *V* is the solution volume and *m* is the sorbent mass.

Exchange capacity of the samples was also determined for the solutions containing 1 × 10^−5^ and 1 × 10^−3^ M U(VI). The samples were in contact with the solutions for 24 h, the ratio of the solid and liquid is mentioned above. After sorption from the 1 × 10^−3^ M solution, some ion-exchangers were regenerated consequentially with deionized water, 1 M Na_2_SO_4_, and 1 M H_2_SO_4_ (the volume of each liquid was 50 cm^3^). The regeneration degree was calculated as $\frac{VC}{Am}$, where *C* is the effluent concentration.

## Results

### Features of ZHP Precipitation in Ion Exchange Matrix

When precipitation of insoluble *CatAn* compound occurs in ion-exchanger, dissolution of small particles and their reprecipitation on larger particles is advantageous from a thermodynamic point of view. Gibbs energy of the system reduces due to decrease of the particle surface. The Ostwald-Freundich equation [51] reflects the effect of particle size on solubility:

1 $$ \ln \frac{{\overline{C}}_{C atAn}}{C_{C atAn,\infty }}=\frac{\beta {V}_m\sigma}{RTr}. $$

Here, ${\overline{C}}_{C atAn}$ is the concentration of dissolved compound in the ion-exchanger, *C*_CatAn,∞_ is the concentration of saturated solution (in the case of ZHP, the ${\overline{C}}_{C atAn}$ and *C*_CatAn,∞_ values are extremely low), *β* is the shape factor of particles, *V*_*m*_ is the molar volume of the compound, *σ* is the surface tension of the solvent, *R* is the gas constant, *r* is the radius of incorporated particles.

For simplification, we can assume a charge number of one both for cations and anions. Thus, ${\overline{C}}_{\mathrm{Ca}\mathrm{tAn}}=\left[\overline{\mathrm{Ca}{\mathrm{t}}^{+}}\right]=\frac{K_{\mathrm{sp}}}{\left[\mathrm{A}{\mathrm{n}}^{-}\right]}$, here, the square brackets correspond to equilibrium concentration, *K*_sp_ is the product solubility. Under excess of a precipitant containing An anions (for instance, HAn acid), it is valid:

2 $$ \left[ A{n}^{-}\right]={C}_{HAn}-\frac{\left[\overline{ C a{ t}^{+}}\right]{V}_i}{V_{HAn}}, $$

where *V*_*i*_ and *V*_*HAn*_ are the volumes of ion-exchanger and acid, respectively, *C*_HAn_ is the initial acid concentration. $\left[{\overline{\mathrm{Cat}}}^{+}\right]= A+{\left[{\overline{\mathrm{Cat}}}^{+}\right]}_{\mathrm{ad}}$, where *A* is the capacity of the ion-exchanger, ${\left[{\overline{\mathrm{Cat}}}^{+}\right]}_{\mathrm{ad}}$ is the concentration of additionally sorbed cations, which are present in the ion-exchanger before precipitation. Thus:

3 $$ {\overline{C}}_{C atAn}=\frac{K_{sp}}{C_{HAn}-\frac{\left( A+{\left[ Ca{t}^{+}\right]}_{ad}\right){V}_i}{V_{HAn}}}, $$

Taking formula (1) into consideration, it is possible to obtain:

4 $$ r=\frac{\mathit{\mathsf{\beta}}{\mathit{\mathsf{V}}}_{\mathit{\mathsf{m}}}\mathit{\mathsf{\sigma}}}{RT \ln \frac{K_{sp}}{C_{C atAn,\infty}\left({C}_{HAn}-\frac{\left( A+{\left[ Ca{t}^{+}\right]}_{ad}\right){V}_i}{V_{HAn}}\right)}}. $$

The particles of a larger size are stable thermodynamically, smaller particles are dissolved and reprecipitated.

As follows from Eq. (4), smaller particles will be formed in the resin with higher exchange capacity. Increasing in concentration of salt, which is used for impregnation of the ion-exchanger before precipitation (increase of ${\left[{\overline{\mathrm{Cat}}}^{+}\right]}_{\mathrm{ad}}$), and decreasing in acid (precipitator) concentration also cause precipitation of smaller particles inside the polymer.

Earlier the effect of molar volume of the insoluble compound on particle size has been found [54]: hydrated zirconium dioxide forms smaller particles than ZHP, which is characterized by higher *V*_*m*_ value. This effect is observed for precipitation in inert polymer. The influence of temperature is considered in [42].

### Morphology of Ion-Exchangers

**Fig. 1:**
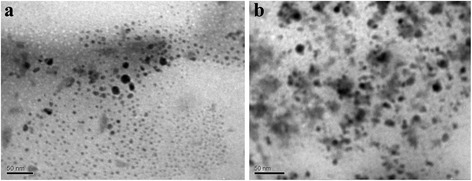
ZHP nanoparticles incorporated into *Dowex HCR-S* (sample *CR-1*, **a**) and *Dowex WX-2* (sample *CR-2*, **b**)

According to data of the producing company, the values of ion exchange capacity are 1.8 and 0.6 mmol cm^−3^ for *Dowex HCR-S* and *Dowex WX-2* resins, respectively. Since the ZrOCl_2_ solution containing soluble zirconium hydroxocomplexes is strongly acidic, only a part of functional groups of the resins is involved into ion exchange during impregnation. Nevertheless, exchange capacity of *Dowex HCR-S* is expected to be higher than that for the flexible resin under these conditions. According to Eq. (4), smaller particles are formed in *Dowex HCR-S* resin (compare *CR-1* and *CR-2* samples, Fig. 1). Sizes of nanoparticles are 4–20 nm. Non-aggregated nanoparticles dominate in *CR-1*, mainly aggregates (up to 50 nm) are seen on the image for *CR-2.* Larger aggregates are formed preferably in the flexible resin evidently as a result of reprecipitation.

Porous structure of gel-like ion exchanges provides location for incorporated particles. The structure, which is formed during swelling, involves clusters (up to 20 nm) and smaller channels between them [38–42, 55–57], where functional groups are located. Larger pores (several tens and even hundreds of nanometers) are voids between gel regions. Hydrophobic fragments of hydrocarbonaceous chains are placed there. At last, pores of micron size are related to structure defects.

As shown previously [39, 40, 42], single nanoparticles are located in clusters and channels, aggregates can be placed in regions between gel fields. The particles are stabilized by pore walls, which prevent further aggregation. Formation of small aggregates is evidently caused by dissolution of nanoparticles in clusters and channels and reprecipitation in regions between gel fields. Precipitation of the inorganic constituent in pores, which exist only in swollen ion-exchanger, increase size of air-dry grains (see Table 1). The grain size of the pristine *Dowex HCR-S* resin was 0.27 mm.

**Fig. 2:**
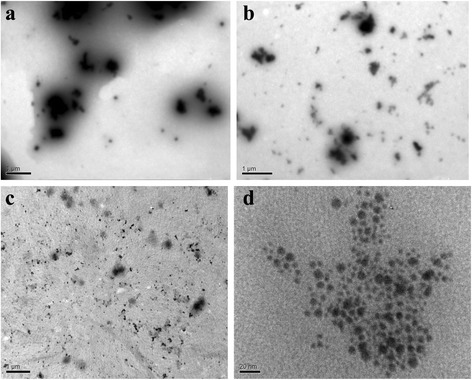
TEM images of *CR-1* (**a**), *CR-3* (**b**), and *CR-7* (**c**, **d**) samples

Other factor determining size of incorporated particles is the amount of additionally sorbed electrolyte. The ${\left[{\overline{\mathrm{Cat}}}^{+}\right]}_{\mathrm{ad}}$ value can be controlled by washing (removal of additionally sorbed electrolyte, which was used for immersion of the ion-exchanger) on the one hand and by regulation of its initial concentration on the other hand. Figure 2 illustrates TEM images for the *CR-1*, *CR-3*, and *CR-7* samples*.*

The particles, size of which is larger than 1 μm, are seen in the image of the *CR-1* sample (no removal of additionally sorbed ZrOCl_2_ during modification). Large aggregates are evidently placed in structure defects. Removal of additionally sorbed electrolyte causes a decrease of particle size. The aggregates (up to 500 nm) are evidently located in voids between gel regions. At last, small particles (<100 nm) dominate in the *CR-7* sample, which was obtained by immersion with low concentrated ZrOCl_2_ solution. A size of non-aggregated nanoparticles is 2–10 nm.

**Fig. 3:**
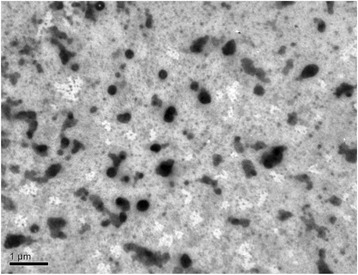
TEM image of the *CR-5* ion-exchanger

Decreasing in the precipitator (H_3_PO_4_) concentration reduces size of aggregates as seen from TEM image for the *CR-5* ion-exchanger (Fig. 3, compare with Fig. 2a). This is in accordance with Eq. (4).

### State of Phosphorus in Incorporated ZHP

**Fig. 4:**
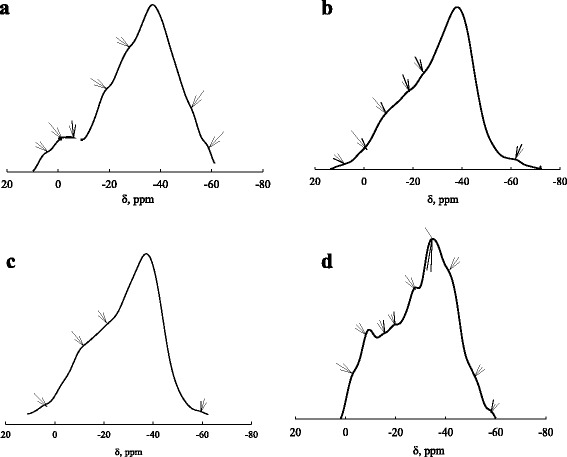
NMR ^31^P spectra for *CR-3* (**a**), *CR-4* (**b**), *CR-5* (**c**), and *CR-6* (**d**)

As shown for individual ZHP ion-exchangers, NMR ^31^P spectra show two signals [41, 48]. It is similar also for ZHP particles in ion exchange resins [41] and membranes [58], when the inorganic constituent is in form of aggregates or agglomerates of nanoparticles [41]. These signals are attributed to hydrophosphate and dihydrophosphate groups. In the case of the samples containing non-aggregated nanoparticles, additional signals are observed (Fig. 4).

The spectra are simplified with a decrease of the acid (precipitator) concentration (transition from *CR-3* to *CR-5*). Alternately, the spectra become more complex, when ZrOCl_2_ concentration decreases (compare data for *CR-3* and *CR-6*). This is probably caused by H_3_PO_4_ capsulation with ZHP nanoparticles. No leaching of acid occurs during washing of the samples with deionized water. On the other hand, formation of polyphosphate in nanoreactors (clusters and channels) is possible during precipitation. Detailed analysis of the spectra is outside this work. Increase of intensity of shoulder of the signal in weak field for the CR*-3–CR-5* samples is observed. It means that the content of –OPO_3_H_2_ groups becomes higher.

When the acid concentration was lower than 0.3 M, no signals of phosphorus were detected (*CR-7* and *CR-8*). It means that the ZHP content is below the detection limit.

### Testing of the Samples

**Fig. 5:**
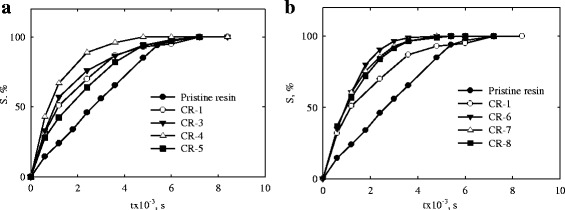
Sorption of U(VI) compounds over time. Here pristine resin is *Dowex HCR-S* (**a**, **b**). Other samples are: *CR-1* (**a**, **b**), *CR-3*, *CR-4*, *CR-5* (**a**), *CR-6*, *CR-7*, *CR-8* (**b**)

Figure 5 illustrates degree of U(VI) sorption over time. As seen, the inorganic particles accelerate sorption (compare the data for the pristine resin and modified ion-exchangers) despite enlargement of grains after modification (see Table 1). The nanoparticles in clusters and channels screen a part of strongly acidic functional groups of the polymer matrix. Since ZHP is a weakly acidic ion-exchanger, only a part of its functional groups is involved into ion exchange. Thus, the acceleration of the process can be explained by an increase of a distance between functional groups of the polymer. Moreover, transformation of porous structure of the polymer constituent could be assumed similarly to [39–42]. However, additional investigations are needed to confirm this assumption.

## Discussion

The rate of sorption decreases in the order: *CR-4 > CR-3 > CR-1 > CR-5.* In general, diminution of size of the incorporated particles accelerates sorption. This tendency is also seen for other samples (*CR-6* > *CR-7* > *CR-8* > *CR-1*).

The models of film and particle diffusions [59], chemical reactions of the pseudo-first [60] and pseudo-second order [61] were used. As found, the *CR-1* and *CR-3*−*CR-5* composites obey the model of the pseudo-first order:

5 $$ \ln \left({A}_{\infty }-{A}_t\right)= \ln {A}_{\infty }-{K}_1 t. $$

**Fig. 6:**
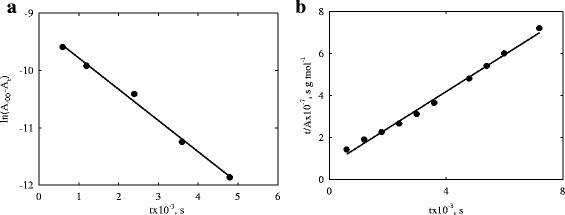
Models of chemical reaction of pseudo-first (**a**) and pseudo-second order (**b**) applied to U(VI) sorption on the *CR-1* and *CR-6* samples

At the same time, the model of the pseudo-second order

6 $$ \frac{t}{A}=\frac{1}{K_2{A}_{\infty}^2}+\frac{1}{A_{\infty }}\cdot \mathit{\mathsf{t}}. $$

can be applied to *CR-6*-*CR-8* samples. Here, *A*_*t*_ and *A*_∞_ are the capacity after certain time and under equilibrium conditions, respectively, *K*_1_ and *K*_2_ are the constants. The results for some samples are given in Fig. 6, the data are summarized in Tables 2 and 3.

**Table 2:** Models of chemical reaction of the pseudo-first order

Sample	*A*_∞_ × 10^4^, mol g^−1^		*K*_1_ × 10^4^, s^−1^	*R* ^2^
	Experimental	Calculated from Eq. (5)		
*CR-1*	1.00	0.95	5.0	0.993
*CR-3*	1.00	0.97	5.1	0.991
*CR-4*	1.00	0.95	8.9	0.999
*CR-5*	1.00	1.07	5.1	0.995

**Table 3:** Models of chemical reaction of the pseudo-second order

Sample	*A*_∞_ × 10^4^, mol g^−1^		*K*_2_, g mol^−1^s^−1^	*R* ^2^
	Experimental	Calculated from Eq. (6)		
*CR-6*	1.00	1.17	9.02	0.985
*CR-7*	1.00	1.19	7.94	0.990
*CR-8*	1.00	1.19	7.47	0.992

Thus, sorption rate is determined by size of incorporated ZHP particles on the one hand and by interaction of sorbed UO_2_^2+^ ions with functional groups of the inorganic constituent on the other hand. If the interaction is complex formation similarly to [48], mainly –(O)_2_PO_2_H groups are involved to sorption (*CR-1*, *CR-3*, *CR-4*, and *CR-5*). When ZHP is precipitated with considerable excess of H_3_PO_4_, significant amount of –OPO_3_H_2_ groups is formed (compare Fig. 4a and d). In this case, UO_2_^2+^ ions interact with them, since these groups are more acidic. However, deposition of insoluble U(VI) compounds is also possible [46]. Detailed investigation of sorption mechanism is a task of future investigations.

Sorption capacity, which is reached in solutions of various concentrations, is given in Table 4 for some samples. Comparing with the pristine resin, the ion-exchangers are characterized by higher exchange capacity. The highest *A*_∞_ values for less and more concentrated solutions were found for the sample containing small aggregates (*CR-7*). In opposite to the sample containing particles of micron size, lower amount of aggressive reagent (H_2_SO_4_) is necessary for regeneration.

**Table 4:** Sorption capacity towards U(VI) and regeneration of the samples

Sample	*A*_∞_ × 10^4^, mol g^−1^		Regeneration degree, %			
	*C*_*i*_ = 1 × 10^−5^ M	*C*_*i*_ = 1 × 10^−3^ M	Water	Na_2_SO_4_	H_2_SO_4_	Total
Pristine resin	0.042	4.00	2	60	38	100
*CR-3*	0.099	3.95	2	15	83	100
*CR-7*	0.098	5.07	2	24	74	100

## Conclusions

A size of ZHP particles incorporated into ion-exchange polymers can be controlled during modification procedure by regulation of concentration of ZrOCl_2_ and H_3_PO_4_ solutions as well as by removal of additionally sorbed ZrOCl_2_. Decrease of the concentration allows us to obtain small particles (both non-aggregated nanoparticles and their aggregates) and avoid formation of agglomerates of micron size. Complex structure of NMR ^31^P spectra has been found. This can be caused by H_3_PO_4_ capsulation in clusters and channels of the polymer on the one hand and by polyphosphate formation on the other hand.

Testing of the samples shows faster sorption of U(VI) cations on the modified sample comparing with the pristine resin. The exchange is complicated by chemical interaction of sorbed U(VI) ions with functional groups of ZHP: the rate of exchange is described by the models of chemical reaction of the pseudo-first or pseudo-second order. Thus, selectivity of the composites towards U(VI) ions is expected for the solution containing other inorganic ions. The sorbents can be used in acidic media.

Control of particle size by regulation of salt concentration looks preferably since ZHP obtained by this manner accelerates sorption despite initial ZrOCl_2_ content in the resin before precipitation. The ion-exchanger obtained by this manner shows significant exchange capacity in wide interval of U(VI) concentration. The approach developed in this work could be used further for purposeful control of particle size in ion exchange polymers.
